# Biomechanical Analysis of Serious Neck Injuries Resulting from Judo

**DOI:** 10.3390/healthcare9020214

**Published:** 2021-02-16

**Authors:** Tomoyuki Nakanishi, Masahito Hitosugi, Haruo Murayama, Arisa Takeda, Yasuki Motozawa, Masahiro Ogino, Katsuhiro Koyama

**Affiliations:** 1 Department of Legal Medicine, Shiga University of Medical Science, Otsu, Shiga 520-2192, Japan; tom_n1976@icloud.com (T.N.); arisa204@belle.shiga-med.ac.jp (A.T.); 2 Department of Health and Sports Sciences, Premedical Sciences, Dokkyo Medical University School of Medicine, Shimotsuga, Tochigi 321-0207, Japan; hmurayam@dokkyomed.ac.jp; 3 Department of Mechanical and Precision System, Teikyo University, Utsunomiya, Tochigi 320-8551, Japan; motozawa@ucatv.ne.jp; 4 Department of Neurosurgery, Dokkyo Medical University School of Medicine, Shimotsuga, Tochigi 321-0207, Japan; oginom@dokkyomed.ac.jp; 5 Graduate School Department of Interdisciplinary Research, University of Yamanashi, Kofu, Yamanashi 400-0016, Japan; koyama@yamanashi.ac.jp

**Keywords:** cervical spine injury, martial arts, judo, biomechanics, injury prevention

## Abstract

To establish a basis for initial diagnosis and for proposing preventive measures for the serious neck injuries occasionally experienced by judo practitioners, the biomechanical mechanisms of these injuries were analyzed. Two male judo experts repeatedly threw an anthropomorphic test device (POLAR dummy) using three throwing techniques (*Seoi-nage*, *Osoto-gari*, and *Ouchi-gari*). The dummy’s kinematic data were captured using a high-speed digital camera, and the load and moment of the neck were measured with load cells. The neck injury criterion (*N*_ij_) and beam criterion were also calculated. In *Seoi-nage*, the anterior and parietal regions of the dummy’s head contacted the tatami (judo mat). Subsequently, most of the body weight was applied, with the neck experiencing the highest compression. However, in *Osoto-gari* and *Ouchi-gari*, the occipital region of the dummy’s head contacted the tatami. Significantly higher values of both *N*_ij_ (median 0.68) and beam criterion (median 0.90) corresponding to a 34.7% to 37.1% risk of neck injury with an abbreviated injury scale score ≥2 were shown in *Seoi-nage* than in either *Ouchi-gari* or *Osoto-gari*. In judo, when thrown by the *Seoi-nage* technique, serious neck injuries can occur as a result of neck compression that occurs when the head contacts the ground.

## 1. Introduction

Sports injuries are a major public health problem. Judo, which became an Olympic sport for men at the Tokyo 1964 Olympics and for women at the Barcelona 1992 Olympics, is the most popular Japanese martial art (budo) in the world. According to the International Judo Federation, there are more than 40 million judo practitioners in more than 200 countries worldwide [1]. A systematic review suggested that the upper extremities are the most commonly injured body region in competitive judo, followed by the head and neck; the most frequent type of injury is contusion [2]. One recent study in France suggested that the upper limbs were the most commonly injured body region, followed by lower limbs and the cervical spine, in judo competitions [3]. Another recent study (in Japan) found that the head and neck were the most frequent regions of minor injury, followed by upper limbs, and the upper limbs were the most frequent regions of major injury, followed by the head and neck [4]. However, though head and neck injuries are not common (incidences of 2.44 and 1.47 cases per 100,000 judo players per year, respectively), they tend to be more serious than injuries in other body regions [5]. Most injuries occur during standing fighting, throwing an opponent, or being thrown [2]. The All Japan Judo Federation, in which most instructors and participants at judo clubs, schools, and company teams are registered, established the System for Compensation for Loss or Damage, which records death or severe residual disability among judo players. The accident reports provided to the All Japan Judo Federation’s System for Compensation for Loss or Damage were analyzed [5]. From 2003 to 2010, 72 accidents were reported including 30 severe head injuries and 19 severe neck injuries (18 cervical spine injuries and 1 atlantoaxial subluxation). In these serious cases, clinicians must quickly assess and adequately manage the injury to prevent negative outcomes. Therefore, it is necessary to elucidate the mechanisms of severe head and neck injuries during judo.

A biomechanical examination of the mechanisms of head injuries during judo and proposal of effective preventive measures have previously been performed [6,7,8,9,10]. However, no studies have been performed to analyze the mechanisms of serious neck injuries in judo. Serious neck injuries reportedly occur most often in practitioners who are executing an offensive maneuver or being thrown [5]. Among the practitioners who sustain such injuries when being thrown, *Seoi-nage* is the most common throwing technique (42.9%) [5], in which the recipient is thrown forward over the thrower’s shoulder. In contrast, serious neck injuries seldom occur when implementing the throwing technique of *Osoto-gari* or *Ouchi-gari*, in which the recipient’s supporting leg is swept by the offender and the recipient falls backward [5].

Therefore, the authors considered that judo practitioners may be more likely to suffer from serious neck injuries when they are thrown by *Seoi-nage* than when they are thrown by other techniques because of greater applied forces at the neck. The main objectives of this study were to biomechanically determine the mechanisms of serious neck injuries in judo practitioners during specific throwing techniques and propose preventive measures for serious neck injuries.

## 2. Materials and Methods

### 2.1. Judo Practice

In this study, the situation in which a practitioner is thrown by three techniques (*Seoi-nage*, *Osoto-gari*, and *Ouchi-gari*) and the head strikes the tatami (judo mat) was created. Two Japanese male judo experts (for *Seoi-nage*: age, 33 years; height, 166 cm; weight, 82 kg; experience, fifth dan, for *Osoto-gari* and *Ouchi-gari*; age, 26 years; height, 177 cm; weight, 90 kg; experience, fifth dan) repeatedly threw an anthropomorphic test device (ATD). Informed consent was obtained from both participants, and the study was approved by the research ethics committee of Dokkyo Medical University School of Medicine (No. 22008).

An ATD is a mechanical model of the human body typically used in vehicle crash testing. Using an ATD, it is possible to obtain mechanical loading parameters at an impact that would be injurious to a person. Among several state-approved models, the POLAR dummy was used in the present study [11,12]. The POLAR dummy (stature, 175 cm; mass, 75 kg) was designed to simulate the kinematics of the human body during vehicle–pedestrian collisions and has high biofidelity. The close similarity of this dummy to humans has been validated with tests using post-mortem human subjects [12]. Because people performing judo experience complex rotations or injuries to the body, the POLAR dummy is suitable for detecting physical parameters with high reliability. Although the neck angle of the dummy is fixed as straight, this is representative of injured judo practitioners who do not perform break-fall techniques adequately.

At the study site, a 6-cm-thick synthetic sponge consisting of urethane and polyethylene (AM2202; Senoh Corp., Matsudo, Chiba, Japan) was laid on a concrete floor. A tatami (SV230; Hayakawa Textile Industries Co., Ltd., Kashiwara, Osaka, Japan) was then placed on the sponge. This type of tatami has been used at international judo tournaments.

In *Seoi-nage*, the recipient is thrown forward over the thrower’s shoulder, and the anterior to parietal aspects of his or her head strike the tatami. According to the All Japan Judo Federation, severe neck injuries have occurred when throwers have placed their knees on the tatami [13]. Therefore, in this study, the thrower placed both knees on the tatami during implementation. In *Osoto-gari*, the thrower breaks the recipient’s balance toward the recipient’s rear corner and then sweeps the recipient’s leg. In *Ouchi-gari*, the thrower pushes the recipient straight back and then sweeps the recipient’s leg from the inside with his or her own leg; subsequently, the recipient is thrown backward, and the occipital aspect of his or her head strikes the tatami.

The judo experts in the current study threw the ATD five times using the *Seoi-nage* technique and four times each using the *Osoto-gari* and *Ouchi-gari* technique.

### 2.2. Data Acquisition

Six-axial load cells were mounted both at the top (Denton Model B-3454J, Denton, MI, USA) and bottom (Denton Model B-4366J, Denton, MI, USA) of the neck to detect the load and moment around the Y-axis of the neck. This moment corresponded to the anterior–posterior movement of the head. Data were recorded using a high-speed data acquisition system (TDAS G5 system, DTS, Tokyo, Japan) that could sample at 20 kHz and were filtered using a Channel Class 1000 filter. This filtering method corresponded to the international standard for vehicle crash test using the dummy [14]. ATD kinematics data were obtained using a high-speed digital video camera (FASTCOM series, PHOTRON LIMITED, Tokyo, Japan) recording at 1000 frames per second.

### 2.3. Biomechanical Parameters

For quantitative evaluation of neck injuries, the neck injury criterion *N*_ij_ was developed and used in the Federal Motor Vehicle Safety Standard 208 [15,16]. This criterion is based on the correlation of data from dummy tests, cadaver tests, and real-world injuries. The *N*_ij_ criterion was calculated as follows: *N*_ij_ = (*F*_z_/*F*_int_) + (*M*_y_/*M*_int_), where *F*_z_ represents the axial forces in the upper neck (either tension or compression) and *M*_y_ represents the flexion/extension bending moment at the occipital condyles. *F*_int_ and *M*_int_ are critical intercept values used for normalization of differently sized dummies. The *N*_ij_ includes the four neck injury predictors according to the directions of *N*_TE_ (tension–extension), *N*_TF_ (tension–flexion), *N*_CE_ (compression–extension), and *N*_CF_ (compression–flexion). The *N*_ij_ is used to predict upper cervical spine injuries with the intent to assess serious neck injuries with an abbreviated injury scale (AIS) score of 3. An injury threshold value of 1.0 corresponds to a 40% risk of an injury with an AIS score of ≥3 and a 75% risk of an injury with an AIS score of ≥2 [17,18].

The beam criterion (BC) was the injury criterion used for lower neck injuries in the present study. This criterion was also developed mainly by cadaver tests and dummy tests [19]. The BC was calculated as follows: BC = (*F*_z_ / *F*_ZC_) + (*M*_y_ / *M*_YC_), where *F*_z_ is the axial tension or compression force and *M*_y_ is the flexion/extension bending moment at the C7-T1 intervertebral disc. *F*_ZC_ is the critical axial load, and *M*_YC_ is the critical moment. An injury threshold value of 1.0 corresponds to a 50% risk of an injury with an AIS score of ≥2 in the human cervical spine [19].

### 2.4. Statistical Analysis

For the comparison of *N*_ij_ and BC values between throwing techniques, Steel-Dwass nonparametric multiple comparison tests were performed. A *p*-value of <0.05 was considered statistically significant.

## 3. Results

### 3.1. ATD Kinematics

**Figure 1:**
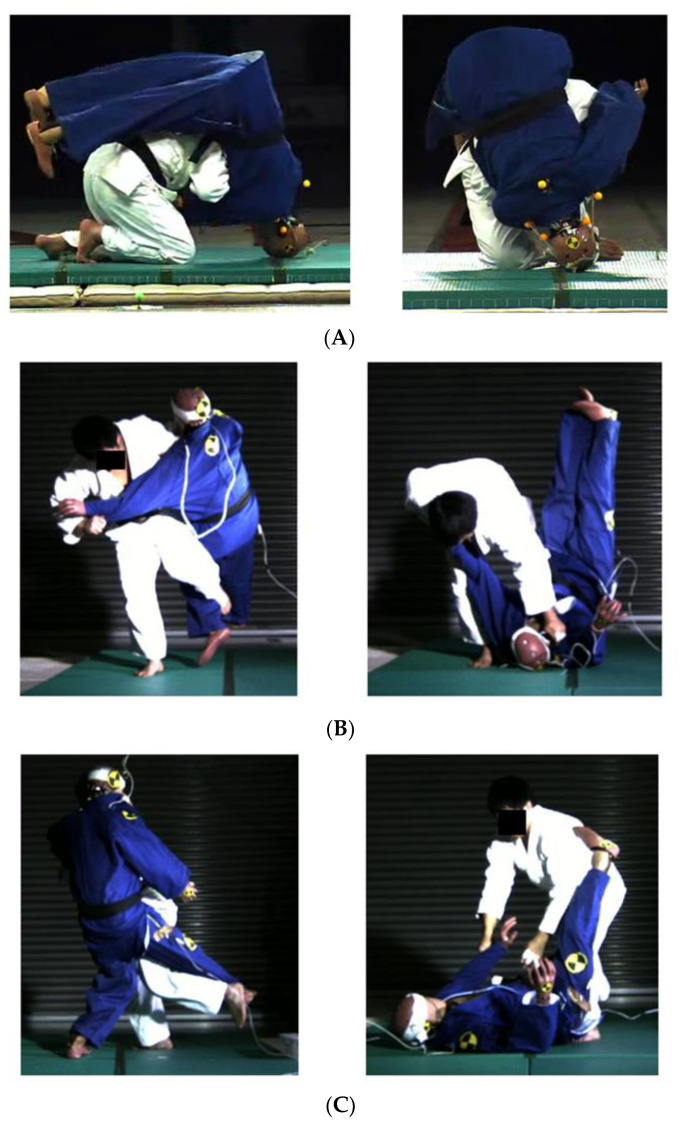
Representative kinematics of the dummy thrown by (**A**) *Seoi-nage*, (**B**) *Osoto-gari*, and (**C**) *Ouchi-gari*.

When *Seoi-nage* was executed, the ATD’s body rode on the thrower’s back, moved forward, and fell to the tatami. The anterior and parietal regions of the head contacted the tatami, and the body then turned forward around the head (Figure 1A). Finally, the ATD landed on its back. In the *Osoto-gari* and *Ouchi-gari* techniques, the ATD fell backward with the occipital area of the head contacting the tatami (Figure 1B,C). In each throwing technique, based on the videos, similar kinematics were observed, and two judo experts (HM and KK) confirmed the repeatability with consistency.

### 3.2. Obtained Values

**Figure 2:**
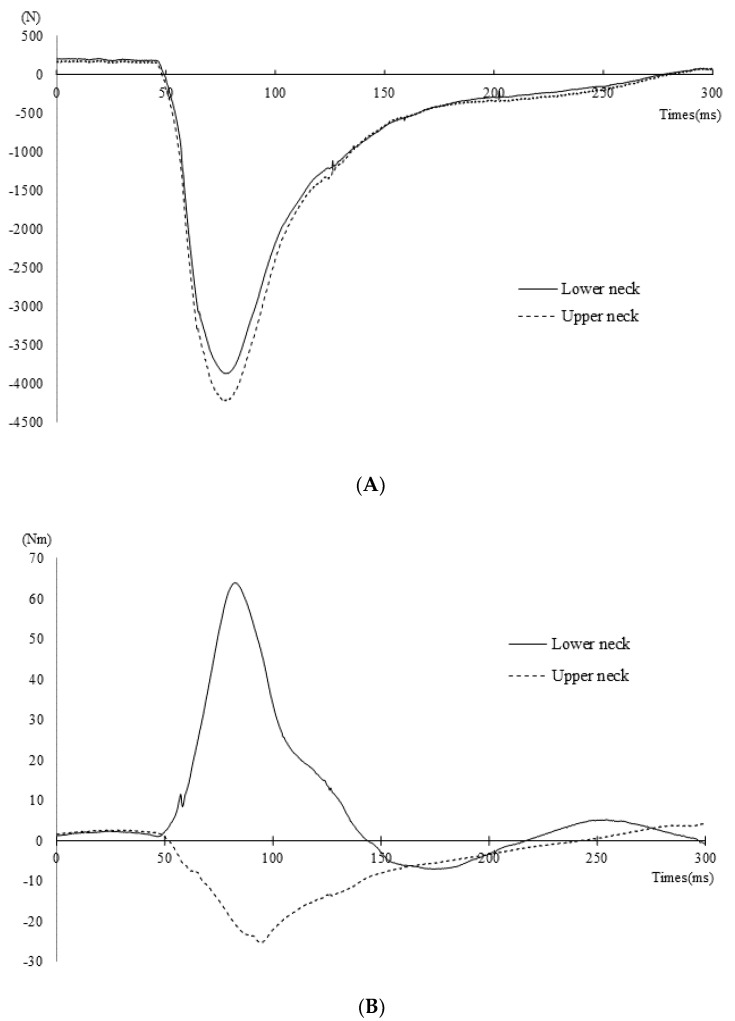
(**A**) Representative time courses of the applied force along with the vertical direction when thrown by *Seoi-nage*. (**B**) Representative time courses of the bending moment along with the anterior–posterior direction when thrown by *Seoi-nage*.

Representative time courses of the *F_z_* and *M_y_* when the ATD was thrown by *Seoi-nage* are shown in Figure 2A,B, respectively. In Figure 2A, a positive *Fz* value indicates tension of the neck and a negative value indicates compression of the neck. The highest negative values were obtained at 76.5 ms in the upper neck (4223.2 N) and at 77.7 ms in the lower neck (3867.6 N). In Figure 2B, a positive *M_y_* value indicates flexion of the neck and a negative value indicates extension of the neck. Shortly after head contact, the highest moments were obtained: 64 Nm was obtained in the lower neck at 82.5 ms, and immediately thereafter, −25.4 Nm was obtained in the upper neck at 94.0 ms. These values indicate that the upper neck was extended and the lower neck was compressed.

### 3.3. N_ij_

**Figure 3:**
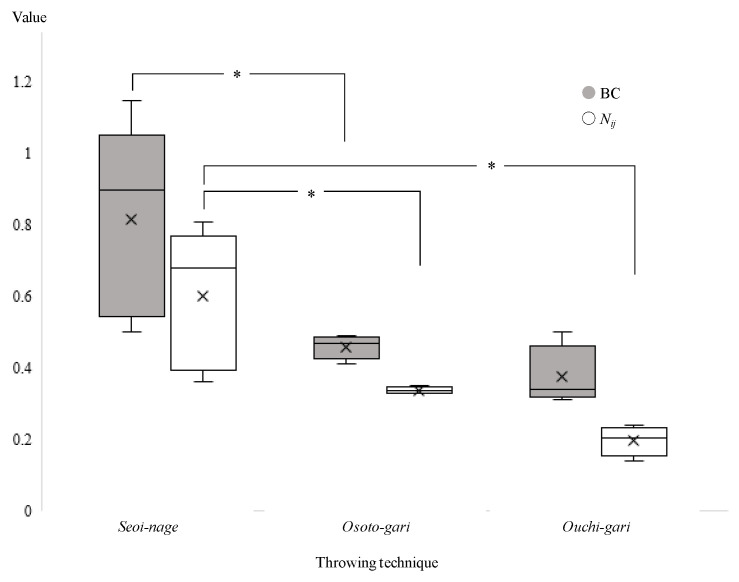
Comparison of neck injury criterion (*N_ij_*) and beam criterion (BC) values among the three throwing techniques. Statistical significance: * *p* < 0.05; Steel-Dwass method.

The *N*_ij_ was calculated from the obtained values in the upper neck of each participant. The four neck injury predictors of the *N*_ij_ (*N*_TE_, *N*_TF_, *N*_CE_, and *N*_CF_) are summarized in Table 1. In the first and second throws using the *Seoi-nage* technique, the highest *N*_ij_ value was shown in *N*_CF_, corresponding to compression–flexion injury of the neck. In the other throws, however, the highest values were shown in *N*_CE_, corresponding to compression–extension injury of the neck. When using the *Osoto-gari* technique, the *N*_CF_ and *N*_CE_ values were similar, and they were markedly smaller than those in the *Seoi-nage* technique. When using the *Ouchi-gari* technique, the values were smaller than when using the other two techniques. In each test, the highest value among *N*_TF_, *N*_TE_, *N*_CF_, and *N*_CE_ was defined as the representative *N*_ij_ value. The representative *N*_ij_ values were compared among the three throwing techniques (Figure 3). The representative *N*_ij_ value of *Seoi-nage* was significantly higher than that of *Ouchi-gari* or *Osoto-gari* (median and interquartile range 0.68 (0.43, 0.73), 0.21 (0.19, 0.22), and 0.34 (0.33, 0.34), respectively; *p* < 0.05).

### 3.4. BC

The calculated values of BC ranged from 0.50 to 1.15 in *Seoi-nage*, from 0.41 to 0.49 in *Osoto-gari*, and from 0.31 to 0.50 in *Ouchi-gari*. BC values were compared among the three throwing techniques (Figure 3). The BC value for *Seoi-nage* was significantly higher than that for either *Ouchi-gari* or *Osoto-gari* (median and interquartile range 0.90 (0.59, 0.95), 0.34 (0.33, 0.35), and 0.47 (0.45, 0.48), respectively; *p* < 0.05).

## 4. Discussion

The purposes of this study were to clarify the mechanisms of serious neck injuries experienced by judo practitioners biomechanically and propose preventive measures. When thrown by the *Seoi-nage* technique, serious neck injuries can occur as a result of neck compression that occurs when the head contacts the ground. To prevent serious neck injuries, effective education of break-fall technique might be required for avoiding the head contact.

Although serious injuries account for only 2% to 4% of all neck injuries, many devastating and costly injuries involving the cervical spine may occur, and some are life-threatening [20]. While injuries to the cervical spine can result from any activity, sports account for a significant portion of injuries to the cervical spine. Because considerable variation exists in sports-related cervical injury, mainly because of differences in playing manners, technical methods, the proficiency and degree of participants, and the contact forces in each competition, it is important to develop effective preventive measures specific to all participants. In addition, the behavior of the cervical spine is nonlinear, rate-dependent, and highly sensitive to the conditions imposed by the loading environment [21]. Certain mechanical determinants, such as the load position, load direction, and magnitude and duration of loading, influence the occurrence of cervical injuries; thus, biomechanical analyses are indispensable for clarifying the mechanism of cervical injuries.

The Japanese Ministry of Education, Culture, Sports, Science, and Technology mandated instruction in Japanese martial arts, including judo, for junior high school students beginning in 2012. Increasing numbers of young practitioners have since been participating in judo. Among the 19 serious neck injuries reported in Japan to date, young practitioners aged <20 years accounted for 57.9% [5]. Therefore, effective preventive measures with clearly detailed mechanisms of serious neck injuries must be established to maintain the practitioners’ safety and eliminate the concern about neck injuries resulting from judo. Because judo is a martial art involving the use of quick throws, it is difficult to determine how and when a serious injury will occur. For analyses of sports injuries, evaluating the kinematics of players is important [22]. Therefore, the present biomechanical method, which recreates the playing situation and analyzes the physiological parameters, is suitable for this investigation. To prevent catastrophic neck injuries in judo practitioners, we biomechanically analyzed several series of values using an ATD.

The results showed significantly higher values of both the *N*_ij_ and BC in *Seoi-nage* than in *Ouchi-gari* or *Osoto-gari*. This finding can be explained by the difference in the kinematics of recipients in whom the frontal to parietal region of the head contacts the tatami in *Seoi-nage* and in whom the occipital region of the head contacts the tatami in *Osoto-gari* or *Ouchi-gari*. Because the anterior–parietal head contacts the tatami and the body is rotated around the head in *Seoi-nage*, most of the body weight is applied on the neck with the highest compression. The highest extension and flexion values were recorded in the compression–extension in three trials and the compression–flexion in two trials. This difference was caused by the attack position and the neck flexion angle at the time of attack. Thereafter, the neck moment around the Y-axis was generated. Therefore, information from the time of injury may help clinicians consider the mechanism of injury and contribute to the diagnosis of neck injuries; a neck injury should be suspected if the practitioner has been thrown by *Seoi-nage* rather than by *Osoto-gai* or *Ouchi-gari*.

The biomechanical parameters used in this study were the *N*_ij_ and BC. Although the monitoring region and formula were different, similar results were obtained for both parameters. The median representative *N*_ij_ value of *Seoi-nage* was 0.68, which corresponded to a 34.7% risk of an injury with an AIS score of ≥2 [18,19]. Additionally, the median BC value for *Seoi-nage* was 0.90, which corresponded to a 37.1% risk of an injury with an AIS score of ≥2 [18,19]. The detailed background characteristics of serious neck injuries, including the impact site of the head and the kinematics of the opponent and recipient, have not been clarified. Our method presented herein seems to be among the best in this research field at present. These results will be confirmed, however, if more precise simulations are performed with higher-resolution videos or more advanced sensing technology.

To prevent serious neck injuries, contact with the recipient’s head should be avoided. If the recipient rotates his or her body while flexing the neck and trunk, the practitioner may land on their back. This is in accordance with the break-fall technique, termed ukemi in judo. This technique requires that the thrown practitioner lands on their back while flexing their head and neck and extending their arms horizontally, which prevents serious head contact with the tatami. In competitions, even well-experienced judo practitioners may not avoid severe head or neck injuries. However, the analysis of kinematics of the break-fall motion suggested that effective education of break-fall technique might lower the risk of judo-related head injuries in novice judo players [23]. Ukemi is also required to prevent serious neck injuries, especially for novice judo practitioners, when being throwing forward. More than half of the serious neck injuries in judo practitioners to date occurred in young or inexperienced practitioners aged <20 years who might not have had adequate break-fall skills [5]. The need for young or inexperienced practitioners to master ukemi before participating in throwing technique practice is emphasized. Because correct ukemi prevents high head accelerations [10], measurement of head acceleration would be useful for evaluating acquired break-fall technique skills in judo practitioners.

Our study has some limitations. First, the ATD’s neck angle was fixed even though the contact point of the head and the applied acceleration depend on the degree of neck flexion. However, the aim of this study was to recreate catastrophic neck injuries by judo, and the reliability of the study is adequate because most practitioners who have sustained severe head or neck injuries were not considered to have enough neck flexion. Further research using a dummy with a flexed neck angle is required to evaluate the effects of neck flexion on preventing severe neck injuries. Second, a few experimental trials were repeated. However, our throwers were judo experts with high-level skills (fifth dan) and reproduced almost identical throwing techniques as confirmed in the recorded videos. Because our obtained values revealed only small deviations, we believe the results to be highly reliable. Third, the occurrence of severe neck injuries depends on the speed of the maneuver. In this study, because judo experts with high-level skills threw the ATD, the maneuvers were high-speed, and the head impact occurred in a short duration. Because *Seoi-nage* was performed by sufficiently experienced judo practitioners, this study reflects real-world situations.

## 5. Conclusions

A significantly higher risk of serious neck injuries when the ATD was thrown by *Seoi-nage* than by *Osoto-gari* or *Ouchi-gari* was found. When the recipient was thrown forward over the thrower’s shoulder and attacked with the anterior-parietal regions of the head to the tatami, high compression–extension or compression–flexion forces were subsequently applied to the neck in *Seoi-nage*. The obtained values showed a substantial risk of serious neck injuries. To our knowledge, this is the first study to biomechanically investigate the mechanisms of neck injuries in judo practitioners utilizing the crash test dummy. These findings may help clinicians to consider the mechanisms of injury when making an initial diagnosis if attending to injured judo practitioners. A follow-up biomechanical study of movements among the judo population will be necessary to implement effective prevention measures.

## Figures and Tables

**Table 1:** Results of the neck injury criterion *N*_ij_ by throwing techniques.

Throwing Technique	No	*N*_TF_	*N*_TE_	*N*_CF_	*N*_CE_
*Seoi-nage*	1	0.15	0.05	0.73	0.68
	2	0.16	0.05	0.36	0.26
	3	0.11	0.02	0.08	0.68
	4	0.11	0.02	0.17	0.43
	5	0.09	0.03	0.05	0.81
*Osoto-gari*	1	0.04	0.13	0.31	0.33
	2	0.02	0.02	0.34	0.28
	3	0.11	0.20	0.33	0.32
	4	0	0.01	0.35	0.35
*Ouchi-gari*	1	0.24	0.06	0.17	0.04
	2	0.04	0.09	0.14	0.14
	3	0.06	0.13	0.20	0.10
	4	0.09	0.10	0.21	0.15

TF: tension–flexion, TE: tension–extension, CF: compression–flexion, CE: compression–extension.

## Data Availability

The data presented in this study are available on request from the corresponding author.
